# Exosomes Derived from Bone Marrow Mesenchymal Stem Cells Alleviate Ischemia-Reperfusion Injury and Promote Survival of Skin Flaps in Rats

**DOI:** 10.3390/life12101567

**Published:** 2022-10-09

**Authors:** Qifang Niu, Yang Yang, Delong Li, Wenwen Guo, Chong Wang, Haoyue Xu, Zhien Feng, Zhengxue Han

**Affiliations:** 1 Department of Oral and Maxillofacial-Head and Neck Oncology, Beijing Stomatological Hospital, Capital Medical University, Beijing 100050, China; 2 Department of Oral and Maxillofacial Surgery, Beijing XingYe Stomatological Hospital, Beijing 102600, China

**Keywords:** bone marrow mesenchymal stem cells, exosomes, skin flap, ischemia-reperfusion injury, angiogenesis

## Abstract

Free tissue flap transplantation is a classic and important method for the clinical repair of tissue defects. However, ischemia-reperfusion (IR) injury can affect the success rate of skin flap transplantation. We used a free abdomen flap rat model to explore the protective effects of exosomes derived from bone marrow mesenchymal stem cells (BMSCs-exosomes) against the IR injury of the skin flap. Exosomes were injected through the tail vein and the flaps were observed and obtained on day 7. We observed that BMSCs-exosomes significantly reduced the necrotic lesions of the skin flap. Furthermore, BMSCs-exosomes relieved oxidative stress and reduced the levels of inflammatory factors. Apoptosis was evaluated via the terminal deoxynucleotidyl transferase dUTP nick-end labeling (TUNEL) assay and Western blot analysis of Bax, Bcl-2. Simultaneously, BMSCs-exosomes promoted the formation of new blood vessels in the IR flap, as confirmed by the increased expression level of VEGFA and the fluorescence co-staining of CD31 and PCNA. Additionally, BMSCs-exosomes considerably increased proliferation and migration of human umbilical vein endothelial cells and promoted angiogenesis in vitro. BMSCs-exosomes could be a promising cell-free therapeutic candidate to reduce IR injury and promote the survival of skin flaps.

## 1. Introduction

Free tissue flap transplantation is a classic and important method for the clinical repair of tissue defects caused by trauma, tumor resection, and congenital malformations [1,2,3]. However, during preparation and transplantation, tissue flaps inevitably suffer from varying degrees of ischemia-reperfusion (IR) injury, which affects the success of transplantation [4,5]. Despite several efforts, partial necrosis of flaps remains a common clinical problem [6,7].

During both ischemia and reperfusion progress, multiple pathophysiological events contribute to tissue injury [8]. The underlying mechanisms include calcium overload, oxidative stress, inflammation, mitochondrial dysfunction, endothelial dysfunction, etc. Recognition that multiple local and systemic factors can cause the failure of skin flap transplantation indicates that drugs targeting a single pathophysiological process may not be able to achieve satisfactory therapeutic effects [9].

Exosomes are cell-derived membrane structures with a diameter between 30 and 200 nm and play important roles in a variety of cellular processes [10,11]. They are thought to be essential intercellular communication regulators and participate in multifaceted, dynamical phases of IR injury and tissue regeneration. They help prevent inflammation, decreased apoptosis, enhanced cell viability, migration activities, and tube formation of endothelial cells by transferring bioactive lipids, nucleic acids (mRNAs and microRNAs), and proteins [12]. Compared with traditional mesenchymal stem cell (MSC)-based therapies, exosomes, as a cell-free therapy, have several advantages, such as stable properties, easy storage, low immunogenicity, and cell-free activity, which may offer new approaches for disease treatment [13,14,15,16].

Several studies have shown that BMSCs-exosomes can effectively relieve IR injury of important organs, enhancing angiogenesis and promoting tissue repair and regeneration [17,18,19]. However, only a few studies have indicated the protective efficacy of reinforced activity of MSCs-exosomes against IR injury of tissue flaps [20,21]. In addition, the mechanism of this process is still unclear. In this study, we focused on whether BMSCs-exosomes therapy has a positive effect on IR injury as a potential adjunct to improve flap survival.

## 2. Materials and Methods

### 2.1. Cell Culture

Rat BMSCs (RASMX-01001) and the complete medium (RAXMX-90011) were purchased from Cyagen Biotech Co., Ltd. (Guangzhou, China). Human umbilical vein endothelial cells (HUVECs, Part # FC-0003) and VascuLife^®^ VEGF Cell Culture Media were purchased from Lifeline Cell Technology^®^ (Walkersville, MD, USA). BMSCs and HUVECs were cultured in the medium under a humidified atmosphere with 5% CO_2_ at 37 °C. Cells were used between passages 4 and 5 for the following experiments.

### 2.2. Exosome Isolation and Identification

BMSCs were cultured in DMEM (Hyclone, Logan, UT, USA), supplemented with 10% exosome-free fetal bovine serum (Exo-FBS; System Biosciences, Palo Alto, CA, USA), and 1% penicillin-streptomycin (P/S). Then, the cells were incubated at 3% O_2_ in a three-gas incubator for 48 h. The medium was collected after incubation, centrifuged at 3000× *g* for 15 min, and passed through a 0.22 μm filter to remove dead cells and debris. The clarified conditioned medium was then size fractionated and concentrated through an Amicon^®^ Ultra-15 100 kDa centrifugal filter (UFC9100, Sigma-Aldrich, St. Louise, MO, USA) and centrifugation at 4000× *g* for 20 min. Next, the Exo Quick-TC reagent (EXOTC10A-1, System Biosciences, Palo Alto, CA, USA) was used to isolate exosomes, following the manufacturer’s instructions. The exosome-containing pellets were resuspended in phosphate-buffered saline (PBS) and stored at −80 °C until further use. A bicinchoninic acid assay (BCA) kit (Beyotime, Suzhou, China) was used to assess the total protein concentration of exosomes. Transmission electron microscopy and NanoSight analysis were used to observe the morphology and measure the size of the exosomes. Exosome markers, including Alix, CD9, and CD63, were analyzed via Western blotting.

### 2.3. Exosome Internalization

The exosomes were labeled with DiI membrane dye (C1036, Beyotime, Suzhou, China) and co-cultured with HUVECs for 1 h. Then, the cells were fixed with 4% paraformaldehyde and stained with 4,6-diamidino-2-phenylindole (DAPI; Southern Biotech, Birmingham, AL, USA). The uptake was observed using fluorescence microscopy.

### 2.4. Cell Proliferation Assay

The HUVECs were seeded at 2 × 10^3^ cells/well into 96-well plates and then co-cultured with different concentrations of BMSCs-exosomes (suspended in basal DMEM) under hypoxia condition (with 1% O_2_ in a three-gas incubator) for 6 h followed by reoxygenation for 24 h. A cell counting kit-8 (CCK8, Dojindo, Kumamoto, Japan) was used for the cell proliferation assay. The optical density (OD) value was measured at 450 nm with a microplate reader.

### 2.5. Scratch Wound-Healing Assay

The HUVECs were seeded at 2 × 10^5^ cells/well into 6-well plates and when they reached a confluence of 80%, scratch wounds were made using a 200 μL micropipette tip. After washing each well twice with PBS, basal DMEM containing exosomes at a final concentration (0, 20, and 200 μg/mL) was added to each well. For each scratch, images of three fields of view were taken at 0, 12, and 24 h. ImageJ software (NIH, Bethesda, MD, USA) was used to measure the residual fractional wound area.

### 2.6. Tube Formation Assay in Matrigel

The HUVECs were seeded at 5 × 10^4^ cells per well onto a Matrigel (No. 354230, BD Biosciences, Franklin Lanes, NJ, USA)-coated 96-well plate and cultured with basal DMEM in the presence of different concentrations (0, 20, and 200 μg/mL) of exosomes at 37 °C. Each concentration was evaluated in three wells. After incubation for 3 h, tube formation was examined by microscopy. The number of master junctions and total tube length were quantified by randomly selecting three fields per well using the ImageJ software.

### 2.7. Animals and Surgical Procedures

Healthy Sprague Dawley rats (male, 250–300 g, 6–8 weeks old) were provided by SPF Biotechnology Co., Ltd. (Beijing, China). All procedures were approved by the Institutional Animal Care and Use Committee of Beijing Stomatological Hospital, Capital Medical University (Ethical code: No. KQYY-202109-005). The experimental study was based on a free abdomen flap model [22,23]. Under sterile conditions, the rats were anesthetized, and a 3 cm (width) × 5 cm (length) flap was marked on the abdomen. Rats were randomly divided into three groups: Sham, IR+PBS, and IR+Exo. For the latter two groups, microvascular clamps were used to induce ischemic damage for 4 h. Reperfusion of the flap was started from the clamp removal. For the IR+Exo group, 0.5 mL of exosomes (1 mg/mL, suspended in PBS) were injected through the tail vein on the day of surgery and days 1, 3, and 5 post-operation. The IR+PBS group was injected with an equal volume of PBS. On day 7, the flaps were divided into three zones and marked as proximal, intermediate, and distal. The intermediate region was dissected and homogenized for Western blot and histological analyses.

### 2.8. Exosome Labeling and Tracking In Vivo

To confirm the delivery and uptake of BMSCs-exosomes by flaps after IR injury, exosomes were labeled with DiR Iodide (DiIC18(7), 40757ES25, YEASEN Biotech Co., Ltd., Shanghai, China) and injected through the tail vein into experimental animals. Exo-red fluorescence in animals was detected using a Pre-clinical In Vivo Imaging System (IVIS Lumina LT, PerkinElmer, Waltham, MA, USA) at 0.5, 1, 3, and 6 h after reperfusion.

### 2.9. Flap Necrosis Evaluation

On postoperative days 1, 3, 5, and 7, flap necrosis was documented using a digital camera and grossly determined by appearance, color, hair condition, and necrosis (*n* = 6). The percentage of flap necrosis was calculated using the ImageJ software with the following formula: percentage of necrosis = flap necrosis area/total flap area × 100%.

### 2.10. Tissue Histology

On day 7, flap tissues were removed and fixed with 4% paraformaldehyde, embedded in paraffin, and cut into 5-μm-thick slices. Tissue slices were stained with hematoxylin-eosin (H&E) and Masson’s Trichrome staining. A light optical microscope (Olympus, Corp, Tokyo, Japan) was used to examine the tissue slices under 100× and 200× magnification. The severity of inflammation and tissue damage was scored from images of H&E and Masson’s Trichrome staining, based on inflammatory cell infiltration, necrosis, edema, hemorrhage, thrombosis, and collagen deposition [24,25]. Five random areas on each microscopic image were examined to score samples, and the total damage scores were calculated as the sum of the scores obtained for each criterion.

### 2.11. Apoptosis Assay

The terminal deoxynucleotidyl transferase dUTP nick-end labeling (TUNEL) assay was performed using the One-Step TUNEL Apoptosis Assay Kit (C1090, Beyotime, Suzhou, China) to measure the extent of apoptosis. Following the TUNEL reaction, slides were treated with DAPI (F6057, Sigma, St. Louise, MO, USA). The TUNEL-positive cells were counted in six random fields per slide.

### 2.12. Analysis of Superoxide Dismutase (SOD) Activity and Malondialdehyde (MDA) Content

SOD activity and MDA content were analyzed to evaluate the oxidative stress status of the flaps. Tissue homogenate was prepared at 4 °C and centrifuged at 10,000× *g* for 10 min. The supernatant was collected, and the protein content was measured using a BCA kit. SOD activity and MDA content were measured using kits (S0101S, S0131S, Beyotime, Suzhou, China), following the manufacturer’s instructions.

### 2.13. Enzyme-Linked Immunosorbent Assay (ELISA)

Tissues of the flap were homogenized and centrifuged at 14,000× *g* for 15 min. The supernatant of protein was determined using a BCA kit (Beyotime, Suzhou, China). ELISA was performed using commercial kits with antibodies against TNF-α (438204, BioLegend, San Diego, CA, USA) and IL-1β (1310122, Dakewe, Shenzhen, China). The results were assessed using a microplate reader at 450 nm.

### 2.14. Western Blot Analysis

Western blotting was conducted as previously described [26]. The primary antibodies were used as follows: CD63 (Bioss, Beijing, China), CD9, Alix, Bax, Bcl-2, VEGFA (Proteintech Group, Rosemont, IL, USA), CD31 (Abcam, Boston, MA, USA), PCNA (CST, Wellesley, MA, USA), and β-actin (Abclonal, Wuhan, China). The HRP-conjugated IgG secondary antibody was purchased from ABclonal. Protein bands were visualized using ECL substrate (Bio-Rad, Hercules, CA, USA), followed by exposure to an automatic imager (Bio-Rad, Hercules, CA, USA).

### 2.15. Immunohistochemical Analysis

The immunohistochemical assay was performed as previously described [27]. The primary antibodies against VEGFA, CD31, and PCNA were used. The secondary antibodies including HRP-conjugated and Alexa Fluor-conjugated IgG were purchased from Abclonal (Wuhan, China; AS014; AS003) and Thermo Fisher Scientific (Waltham, MA, USA; Alexa Fluor 594, A-21207; Alexa Fluor 488, A-21202).

### 2.16. Statistical Analysis

GraphPad Prism software (version 8.0) was used for data analysis. The data differences among groups were compared using Student’s *t*-tests, one-way or two-way analysis of variance (ANOVA). Differences were considered statistically significant at *p* values < 0.05.

## 3. Results

### 3.1. Exosome Identification and Internalization In Vitro, and Tracking In Vivo

**Figure 1:**
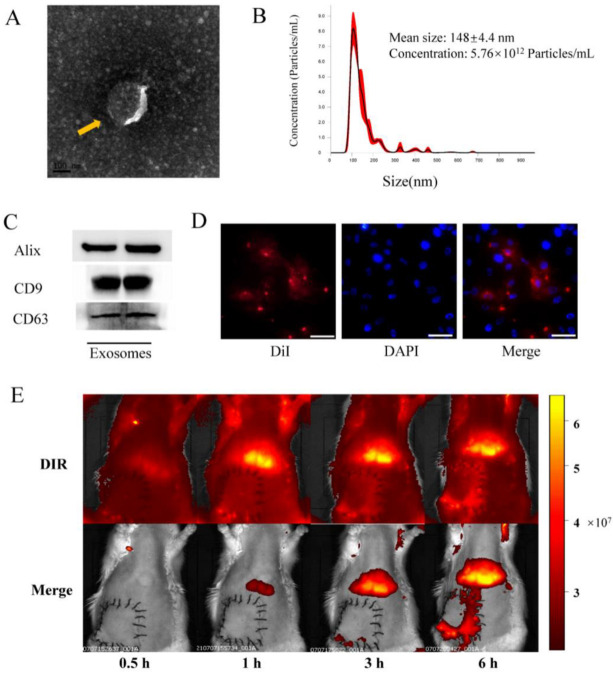
Identification and internalization of BMSCs-exo in vitro. (**A**) Representative images of BMSCs-exo morphology obtained via transmission electron microscopy (yellow arrow, scale bar, 100 nm); (**B**) Size distribution of BMSCs-exo, as determined by NanoSight; (**C**) Identification of exosomes via detection of Alix, CD9, and CD63 using Western blotting; (**D**) DiI-labeled exosomes were detected in HUVECs by confocal fluorescence microscopy (Scale bar, 50 μm); (**E**) In vivo tracking of DiR-labeled BMSCs-exo.

The morphologies and particle sizes of exosomes are shown in Figure 1. The results showed that BMSCs-exosomes displayed a circular and double membrane wrapped shape (Figure 1A). Size analysis further revealed that the average size of exosomes was 148 ± 4.4 nm (Figure 1B). Western blot analysis of BMSCs-exosomes confirmed the presence of exosome markers, including Alix, CD9, and CD63 (Figure 1C). In vitro fluorescence microscopy analysis showed that the DiI-labeled exosomes were taken up by HUVECs, indicating the internalization of BMSCs-exosomes by the cells (Figure 1D). Upon in vivo imaging, the DiR-labeled (red fluorescent) exosomes were found to accumulate in the liver and lungs, with a tendency to aggregate in reperfusion flap tissues after 3 and 6 h, showing homing ability in damaged tissues (Figure 1E).

### 3.2. Exosomes Reduce Necrosis and Histological Lesions of Flaps

**Figure 2:**
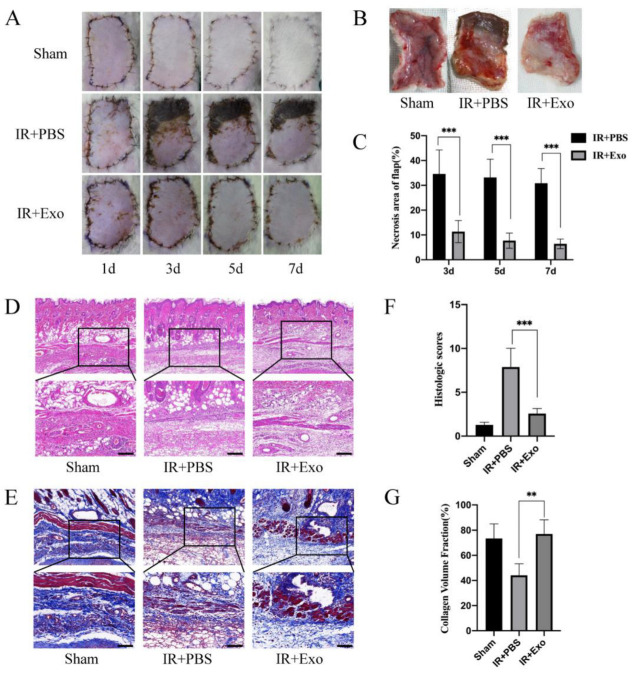
The effects of BMSCs-exo on the ischemia-reperfusion injury flaps’ survival rate and histological lesions. (**A**) Digital photograph of flaps from day 1 to day 7 after surgery; (**B**) The tissue side of flaps on day 7; (**C**) Necrosis rates (necrosis area/flap area) of the IR+PBS and IR+Exo groups, *** *p* < 0.001; (**D**,**E**) H&E and Masson staining of tissue sections of the three groups; (**F**) Histological score of the three groups, *** *p* < 0.001; (**G**) Collagen volume fraction (collagen area/total area) of the three groups, ** *p* < 0.01.

Flaps were observed and photographed on days 1, 3, 5, and 7 after the operation and harvested on day 7. The necrosis rates of skin flaps differed considerably between the IR+PBS and IR+Exo groups. From day 3 onward, circumscriptions between the surviving and necrotic parts became visible in the flaps of the IR+PBS group, while the IR+Exo group was found to have quite a small region of necrosis at the suturing edge (*p* < 0.001; Figure 2A–C). H&E and MT staining revealed distinct pathological changes in the ischemic flap tissue, including increased vacuolar degeneration, inflammatory cell infiltration, and lower density of newly formed vessels (Figure 2D,E). The IR+PBS group showed significantly higher histological scores, compared with the other two groups (*p* < 0.001; Figure 2F). The tissue collagen volume fraction was assessed by MT staining and the fiber area of the IR+Exo group increased significantly compared with that of the IR+PBS group (*p* < 0.01; Figure 2G).

### 3.3. Exosomes Promote Tissue Regeneration and Angiogenesis

**Figure 3:**
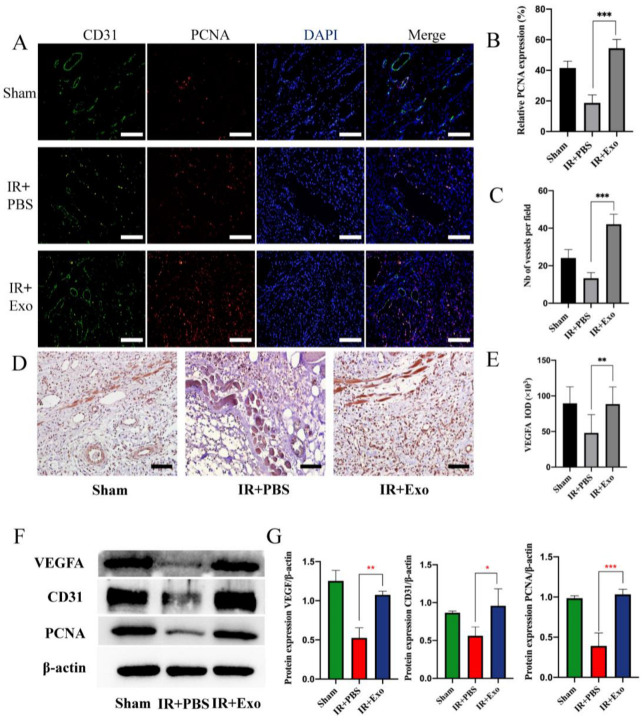
BMSCs-exo promoted tissue regeneration and angiogenesis. (**A**) Co-staining of CD31 (green) and PCNA (red) of the three groups (scale bar, 50 μm); (**B**,**C**) Number of vessels and relative expression of PCNA, *** *p* < 0.001; (**D**) The expression of VEGFA in the three groups detected by immunohistochemistry (scale bar, 50 μm); (**E**) Positive area of VEGFA, ** *p* < 0.01; (**F**) Western blot analysis of VEGFA, CD31, and PCNA expression of the three groups; (**G**) Quantitative analysis of the expression levels of VEGFA, CD31, and PCNA, * *p* < 0.05, ** *p* < 0.01 and *** *p* < 0.001.

Immunofluorescence was used to assess the co-expression of CD31 and PCNA to evaluate the extent of vessel regeneration in the three groups (Figure 3A). The results suggested that BMSCs-exosomes could significantly increase the number of vessels and PCNA-positive cells (*p* < 0.001) (Figure 3B,C). Immunohistochemical analysis showed that exosome injection promoted VEGFA expression in IR flaps (*p* < 0.01; Figure 3D,E). The Western blot analysis showed the same results (*p* < 0.05; Figure 3F,G).

### 3.4. Exosomes Reduce Tissue Apoptosis, Oxidative Stress, and Inflammation of Skin Flap

**Figure 4:**
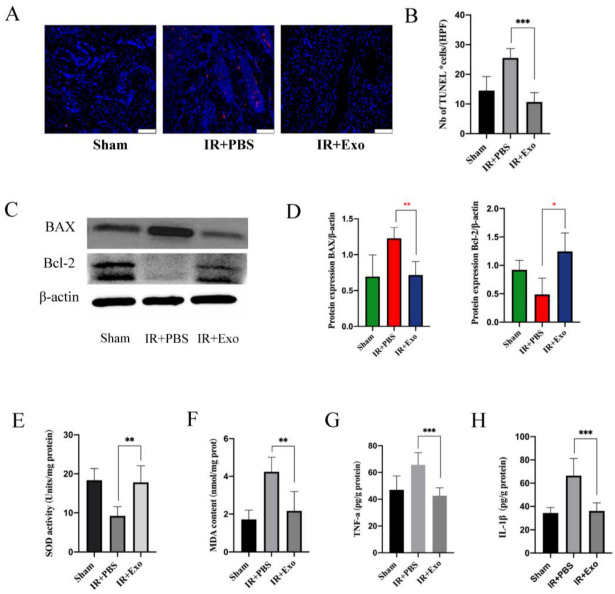
BMSCs-exo reduced the apoptosis of skin flap. (**A**) TUNEL staining (red) of tissue sections of the three groups (scale bar, 50 μm); (**B**) Number of TUNEL positive cells, *** *p* < 0.001; (**C**) Western blot analysis of Bax and Bcl-2 expression in the three groups; (**D**) Quantitative analysis of the expression levels of Bax and Bcl-2, * *p* < 0.05 and * *p* < 0.01; (**E**,**F**) Evaluation of SOD activities and MDA contents of the three groups, ** *p* < 0.01; (**G**,**H**) Expression levels of TNF-a and IL-1β were measured by ELISA analysis, *** *p* < 0.001.

Tissue apoptosis was detected via the TUNEL assay (Figure 4A). The apoptotic cells in the IR+Exo group were remarkably less than that in the IR+PBS groups (*p* < 0.001) (Figure 4B). Subsequently, Western blotting of Bax and Bcl-2 was performed to verify tissue apoptosis (Figure 4C). The relative expression level of the apoptotic protein Bax significantly increased (*p* < 0.01), while the anti-apoptosis protein Bcl-2 level was reduced in the IR+PBS group (*p* < 0.05) (Figure 4D). Collectively, these results suggest that BMSCs-exosomes significantly reduced apoptosis in the flaps that experienced IR injury.

SOD and MDA levels of the flaps were determined to evaluate the effect of BMSCs-exosomes on the extent of oxidative stress, which can significantly affect the survival of flaps. Compared with the IR+PBS group, the IR+Exo group had a high SOD activity and low MDA levels (*p* < 0.01; Figure 4E,F). In addition, cytokine levels were measured in flaps with IR injury to assess the local inflammatory changes. ELISA results showed that the expression levels of IL-1β and TNF-a in the IR+Exo group were significantly lower than those in the IR+PBS groups (*p* < 0.001; Figure 4G,H).

### 3.5. Increased Proangiogenic Activities of Exosomes In Vitro

**Figure 5:**
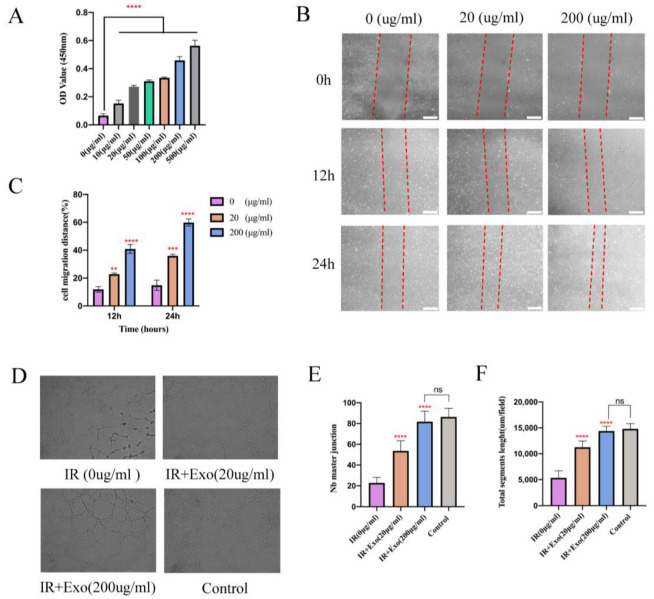
The proliferation, migration, and angiogenesis ability promotion effects of BMSCs-exo on HUVECs in vitro. (**A**) CCK8 test for different concentrations of BMSCs-exo on the HUVECs’ proliferation. Compared to the PBS group, BMSCs-exosomes significantly increased endothelial cell proliferation at 24 h after undergoing hypoxia reoxygenation injury, **** *p* < 0.0001); (**B**) The images of wound healing assay in HUVECs with/without BMSCs-exo for 12 h and 24 h (Scale bar, 100 μm); (**C**) The wound closure rates of HUVECs are presented as migration area/original area, ** *p* < 0.01, *** *p* < 0.001 and **** *p* < 0.0001). (**D**) For capillary network formation assay in vitro, HUVECs were incubated in Matrigel with/without BMSCs-exo. (**E**,**F**) Quantification of capillary network formation of HUVECs, **** *p* < 0.0001.

Since vascularization of ischemic flap tissue is a critical step, we examined the proangiogenic effects of BMSCs-exosomes co-cultured with HUVECs that underwent hypoxia and reoxygenation injury. Proliferation exhibited a marked concentration-dependent enhancement in the groups with exosomes compared to the basal DMEM group (*p* < 0.001; Figure 5A). In the scratch wound-healing assay, the Exo groups migrated noticeably faster compared with that of the basal DMEM group (all *p* < 0.01) (Figure 5B,C). Moreover, a tube formation assay showed that groups with exosomes generated more cord-like structures on Matrigel than in the basal DMEM group (*p* < 0.001) (Figure 5D–F). These results indicate that BMSCs-exosomes enhanced the angiogenesis capacity of hypoxia-reoxygenation vein endothelial cells, as reflected by enhanced proliferation, migration, and capillary network formation in vitro.

## 4. Discussion

Flap transplantation is an essential tissue or organ reconstruction method for cancer surgery or trauma [2]. However, partial necrosis of flaps caused by IR injury remains a common clinical problem that may lead to disastrous consequences, such as secondary surgery and increased financial burden on patients [28]. Thus, there is a need to find an effective way to improve the success rates of flap transplantation. Accumulating evidence indicates that exosomes derived from stem cells offer distinct advantages such as low carcinogenicity, reduced immunity-related risks, and high target specificity, which may provide a promising and safe alternative for tissue regeneration and reconstruction by the application of them, compared with stem cells [29,30].

Oxidative stress, inflammation, apoptosis, and lack of new blood vessels caused by a hypoxia state during ischemia and reperfusion in the transplantation process are the main threats to free flaps [31,32]. Pharmaceutical targeting all these processes can protect skin flaps from partial necrosis. It is well known that exosomes derived from MSCs play important roles in collagen synthesizing and extracellular matrix (ECM) remodeling during the process of tissue regeneration and wound healing [33]. In this study, we assessed the effects of BMSCs-exosomes on flap survival during IR injury. These exosomes attenuated oxidative stress, inhibited apoptosis, promoted angiogenesis, and stimulated tissue healing, thus aiding flap survival. Compared with the control group, flaps treated with intravenous injection of the exosomes showed less necrosis ratio, a more standardized structure with better tissue score and higher content of collagen fibers, reduced cellular vacuolation, and alleviated inflammatory cell infiltration. Furthermore, the results of analyses of MDA content and SOD activity indicate that BMSCs-exosomes may improve skin flap survival by increasing the activity of antioxidant enzymes to reduce oxidative stress. We also found that the exosomes can considerably reduce the expression levels of the inflammatory factors TNF-α and IL-1β. The anti-apoptotic potential of the exosomes was also confirmed by the TUNEL assay and Western blot results of BAX and Bcl-2 expression.

Vascularization of the ischemic flap tissue is a critical step in the process of alleviating IR injury [34]. It is known that many signaling pathways, such as Wnt, may play a central role in vascular development. Recent studies have reported that Wnt signaling activity in target cells can be induced by MSCs-exosomes and play an important role in angiogenesis and tissue repair [35]. It was found that the knockdown of Wnt4 in MSC-exosomes delays the expression of CD31 in vivo and the tube formation of endothelial cells in vitro in a rat skin burn model [36]. Moreover, exosomes derived from adipose mesenchymal stem cells were confirmed to carry matrix metalloproteinases (MMPs), which may promote angiogenesis by facilitating endothelial cell migration [37]. In this study, BMSCs-exosomes promoted the formation of new blood vessels in vivo, as confirmed by the increased expression levels of VEGFA and the fluorescence co-staining of CD31 and PCNA. The exosomes could also promote HUVEC proliferation, migration, and proangiogenic potential in vitro. These findings show that the promotion of tissue angiogenesis may mediate the excellent ability of the exosomes to alleviate tissue injury. However, the mechanisms and intracellular pathways of BMSCs-exosomes in regulating the angiogenesis of flap tissue remain elusive and require further study.

Several studies have shown the tissue-homing ability of exosomes derived from MSCs, which can promote tissue detainment safely and avoid the risks associated with the use of stem cells [38,39]. Exosomes injected into circulation can be cleared nonspecifically under physiological conditions but may gather in tissues with inflammation or other pathological changes [40]. A strong behavior of the uptake of exosomes was confirmed in the ischemic brain tissue in stroke models of rats and rabbits [41,42]. Exosomes in the ischemic area may persist at stable concentrations for more than 24 h, while the concentrations of exosomes in other tissues decrease continuously [43]. The mechanism of the aggregation of exosomes in pathological tissues is still unclear. It may be related to the recognition of specific receptors on exosomes by inflammatory cells or caused by the increased permeability of tissues with injury or inflammation. In our experiment, BMSCs-exosomes were observed specifically targeting flap tissues after IR injury in addition to their distribution in the liver, lung, kidney, or heart, confirming the continuous uptake of exosomes by the flap with injury.

In most studies, exosomes did not show significant cytotoxicity in normal cells, but the side effects following systematic administration still need attention. Therefore, study on the safe dosage and mode of administration to improve the targeting ability and tissue detainment of exosomes is imperative. The therapeutic effect of exosomes was dose-dependent within a certain range in our experiment, and a suitable concentration was chosen by us to ensure safety and therapeutic effects simultaneously. No obvious side effects were found in the rat model in the short term, while further observation is needed to determine whether long-term side effects exist. In recent years, more and more strategies have been developed to achieve more desired outcomes, such as pretreatment or modification of exosomes [44,45]. Moreover, biomaterials such as hydrogel were applicated in exosome delivery or retaining [46,47]. We believe this approach could be a novel solution to the clinical issues associated with IR injury and necrosis of the flap.

## 5. Conclusions

In summary, our research highlights that exosomes derived from BMSCs alleviate the necrosis of the skin flap by relieving oxidative stress, inflammation, and apoptosis in the process of ischemia-reperfusion injury and promote angiogenesis of the flap. Meanwhile, BMSCs-exosomes can enhance the proliferation, migration, and angiogenesis ability of HUVECs. BMSCs-exosomes could be a promising cell-free therapeutic candidate to reduce IR injury and promote the survival of skin flaps.

## Data Availability

Not applicable.
